# Segmentation of Medical Image Using Novel Dilated Ghost Deep Learning Model

**DOI:** 10.1155/2022/6872045

**Published:** 2022-08-12

**Authors:** Marcelo Zambrano-Vizuete, Miguel Botto-Tobar, Carmen Huerta-Suárez, Wladimir Paredes-Parada, Darwin Patiño Pérez, Tariq Ahamed Ahanger, Neilys Gonzalez

**Affiliations:** ^1^Instituto Tecnológico Universitario Rumiñahui, Sangolquí, Ecuador; ^2^Universidad Técnica del Norte, Ibarra, Ecuador; ^3^Eindhoven University of Technology, Eindhoven, Netherlands; ^4^Research Group in Artificial Intelligence and Information Technology, University of Guayaquil, Guayaquil, Ecuador; ^5^Department of Management Information Systems, College of Business Administration, Prince Sattam Bin Abdulaziz University, Al-Kharj, Saudi Arabia; ^6^Instituto de Meteorología, Criisto de La Havana, Cuba

## Abstract

Image segmentation and computer vision are becoming more important in computer-aided design. A computer algorithm extracts image borders, colours, and textures. It also depletes resources. Technical knowledge is required to extract information about distinctive features. There is currently no medical picture segmentation or recognition software available. The proposed model has 13 layers and uses dilated convolution and max-pooling to extract small features. Ghost model deletes the duplicated features, makes the process easier, and reduces the complexity. The Convolution Neural Network (CNN) generates a feature vector map and improves the accuracy of area or bounding box proposals. Restructuring is required for healing. As a result, convolutional neural networks segment medical images. It is possible to acquire the beginning region of a segmented medical image. The proposed model gives better results as compared to the traditional models, it gives an accuracy of 96.05, Precision 98.2, and recall 95.78. The first findings are improved by thickening and categorising the image's pixels. Morphological techniques may be used to segment medical images. Experiments demonstrate that the recommended segmentation strategy is effective. This study rethinks medical image segmentation methods.

## 1. Introduction

Image analysis (image segmentation), and visualisation (machine vision) are all subcategories of digital image processing. In recent years, it has become increasingly difficult to separate organs, disorders, and abnormalities in the medical picture analysis. Medical image segmentation aids in the monitoring of illnesses such as cancer, the modification of medicine doses, and the reduction of radiation exposure. Medical pictures with artefacts make segmentation challenging. These findings were validated using a variety of medical image datasets and segmentation criteria. In contrast to past studies and surveys, the new study looks at the many problems with medical image segmentation as well as some new ways to solve them. Salutations [1, 2] image segmentation divides an input picture into segments that are highly linked with the ROI of the given image. Medical picture segmentation is used to estimate and schedule radiation doses [3]. Image segmentation assists medical picture analysis by emphasising the focus of the image. MRI segmentation methods include recognising brain tumour borders, mammographic mass segmentation, and segmenting pneumonia-affected areas in chest X-rays and CT images. Algorithms have been developed to address the scarcity of medical picture segmentation knowledge [4]. Previous image segmentation models [3, 5] made use of thresholding, edge-based, and region-based methodologies. Pixels were allocated to groups depending on their value range using thresholding. The edge-based technique classifies pixels as edged or nonedged using a filter. Comparable pixels were separated from distinct pixels using the region-based segmentation.

Medical picture segmentation is a time-consuming and demanding process [6]. Image segmentation is usually regarded as the most crucial phase in medical imaging. This is because it computes a ROI using a semiautomatic or fully automated approach. It might be used in medical applications to detect boundaries, tumours, and masses by segmenting an image into portions according to a predefined description of the region of interest. As a result, the image may be broken into smaller sections to facilitate viewing. Clustering algorithms may be used to extract the global attributes of a picture, which can subsequently be used to segment the image. Therefore, the ROI and backdrop may be differentiated more precisely. There are a few medical imaging experts [7]. Over time, deep learning networks have enhanced image, a convolutional neural network classifies pixels in an image by segmenting the picture and then feeding those segments into the network. The network will then partition the image further using the labels it received from the segments. Unlike CNN, other computer systems can process the entire image at once. Once the entire picture has been covered, each future iteration of the mapping process will employ a smaller and smaller “filter” of pixels. Traditional CNNs cannot accommodate a broad range of input sizes because of the way they are built. FCNs analyse data using convolutional layers, and they can handle a wide range of input sizes. As a consequence, they can complete their tasks more rapidly. The last output layer of the image, which has a large receptive field, displays the image's height and breadth. Each class in this layer has the same number of channels as the number of classes in this layer. Convolutional layers give each pixel in an image a category. This makes the image's context, which includes the position of objects. On popular datasets, deep neural networks are believed to be accurate. Picture segmentation may be classified into two types: semantic and instance segmentation. Pixel classification is semantic segmentation. Each object of interest in a photograph is identified and delineated using a computer programme.

Denoising, restoring, and other operations may boost the sharpness of a picture or highlight certain characteristics. Medical pictures are used to segment organs and tissues quantitatively. There are five ways to divide up medical images: threshold-based segmentation, region-growing segmentation, deformation model sectioning, graph theory sectioning, and machine-learning segmentation. Threshold-based medical picture segmentation is included in the first category. It differentiates pixels based on their grayscale values. If the picture quality is good, the target and background are visible, and the border is evident, use OCT images to distinguish between retinal cystic edema and other conditions. The threshold segmentation strategy does not work in this case because there are no obvious low points in the data. Threshold segmentation is good since it makes noise and unevenness into consideration. This strategy is effective for targets that are all connected in the same direction. Because of its sensitivity to noise, the removed component may experience a vacuum or dissociation. In addition to this, further image processing methods must be used [8, 9].

Use a deformation model to segment the medical images. This method is popular because it makes use of regional and border data. The active shape model is the source of today's most extensively used active appearance model (AAM). Combining contours and textures allows for more precise image splitting. The contour detection method may be used to determine the borders of homogenous zones. The approach of “expanding regions,” which assists in the closure of picture contours, can be used to enhance segmentation. Identify growth centres that are attached to non-closed areas and used in the region's expansion split-and-merge procedure. The notion of picking development centres originated here. Stairway closures may be written simply while retaining the original form of the staircase. This method improves accuracy by using past information from training sets (such as cardiac contours) or surfaces with closed or open parameters. The model's applicability is limited by the parameters. A model's accuracy will suffer if its test data differs significantly from its training data. A model is built with the aid of a training set, and its correctness is tested with the help of a test set, also known as a validation set. As a result, the test set does not contain any data points from the training set. A discrete training and validation set is extensively used for each iteration of the technique, as is the norm of splitting data into separate training and validation sets. You may also divide a dataset in half and use one half for training, another for verifying the model, and the third for testing the model. As a result, each half of the dataset may be used for all the three tasks. Unsupervised picture segmentation is a method that changes the game and does not need any more algorithms to be made.

## 2. Literature Review

The number of nodes per pixel in a medical picture may be equal to the pixel count. The graph is segmented to maximise flow and minimise cutting by connecting the foreground and background seed points. Graph theory-based segmentation algorithms such as graph cut and graph search have shown effectiveness in a variety of image modalities [10–12]. A local optimum may be avoided by finding the global optimal solution with the appropriate energy function. Temperature changes might affect the seed point. Traditional medical picture segmentation algorithms have often fallen short of the expectations of the machine learning-based solutions. Boosting-based image segmentation to increase the accuracy of learning algorithms and appropriately coupled prediction functions must be developed [13, 14]. Medical picture segmentation is influenced by the support vector machine (SVM).

However, because of the limited number of kernel functions and the inability to adapt to new scenarios, this technology is inappropriate for widespread usage and commercialization. Third is neural network-based medical picture segmentation [15, 16]. When it comes to medical image segmentation, however, this technique does not capture association and multiscale as well as other algorithms. It is hard to find methods for learning multilayer structures that make the most of spatially related connections [17–19]. Neural networks may reflect human brain processing and learning, as well as human brain input interpretation. Deep learning has been used to separate prostates in magnetic resonance imaging (MRI) [20], the cartilage in the knees [21], ventricular chambers in ultrasonography [22], and tissue in breast imaging [23]. Deep learning has several advantages, but it also has some disadvantages. Neuronal cells must interact in the same way. The human visual neural network is made up of many feedforwards, feedback, and lateral connections [24, 25].

In research [26, 27], feedforward connections are several times more common than feedback connections. A feedforward neural network may be effective in certain sensing applications. It is feasible to employ neural networks as intelligent sensors to more efficiently model and govern the processes. This study focuses on the application of soft sensors in the cane sugar business. A neural network must be trained on historical data before it can be used to anticipate process quality parameters. One of the immediate benefits of making smart sensors is that a neural network can tell exactly what a product's quality is at any given time. The technique's inability to learn and become more resilient in the face of noise remains a concern, as does its poor interpretability and reliance on large amounts of data. A natural feedback control method is the e-feedback neural circuit. More than a feedforward network with extraordinary mapping skills is required to develop higher degrees of artificial intelligence. Feedback is required in tasks such as focused attention, target localization, feature grouping, multitasking, and environmental adaptation. Since this discovery, there has been a surge of interest in feedback convolutional neural networks. Cao comprehends deep learning via the use of CNN. It is used to assess the activity of deep brain neuronal layers. An efficient method for seeing and comprehending neural networks [23, 28, 29] is a more formalised version of that. However, it does not specify how to better organise feedback. The reason for the decreased object identification accuracy has not been explained or optimised. Amir and colleagues discovered that a generic feedback (recurrent neural network) RNN architecture beat a feedforward RNN in trials. Using convolutional neural networks with feedback mechanisms, several well-known scientists have made systems that can classify and identify images [26, 27, 30–33]. Despite these successes, it is still a work in progress, such as better accuracy. This technology is missing a critical feedback mechanism for medical image segmentation.

## 3. Proposed Method

Deep convolutional neural networks face a problem when utilising a target-driven strategy to choose neurons for feedback modification. During the investigation, the reader may choose between two greedy feedback optimization strategies. The convolutional neural network feedback adjustment strategy was developed to govern feedback in deep convolutional networks. The approach is then used to look at the data from the medical image segmentation and make a summary of it. In developing algorithms for medical picture segmentation, the feedback mechanism of a convolutional neural network is discovered and investigated in this study. From the preprocessed image collection, fixed-size sample picture blocks are taken from the trained image collection and used for training. It learns initial parameters for feature learning using unlabeled image block samples and final parameters using labelled image block samples. Using fine-tuned annotated image block samples, convolutional neural networks may be taught to recognise and categorise pictures. Labeled content is added to the binary black-and-white picture first, followed by image block segmentation. Thresholding is a common segmentation technique for distinguishing an item from its surroundings. Thresholding pictures requires comparing the brightness of each image's pixels to a predefined threshold value. When a picture is imported by the programme, it can be separated into two distinct sets of pixels. Pixel intensities in the final image that fall below the threshold are deleted. Pixels whose intensity exceeds the image's threshold combine threshold segmentation with morphological processing before proceeding with additional processing.

**Figure 1:**
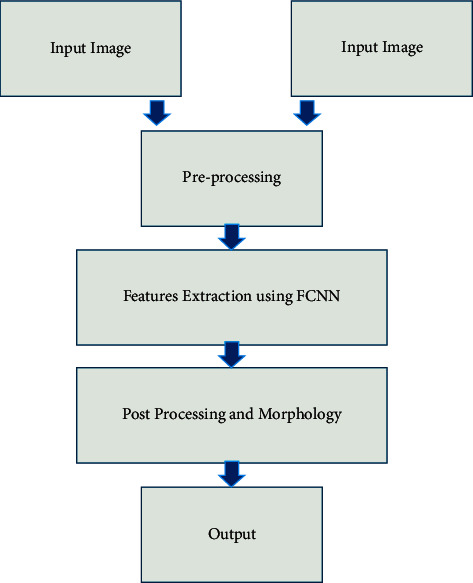
Image segmentation process with FCNN.

First, the Fuzzy Convolution Neural Network (FCNN) is tested at regular intervals. First, gather the test data shown in Figure 1. The purpose of this research is to distinguish medical pictures using classification findings and neighbouring pixel connections. The classification result of the picture block affects the categorization of the centre pixel. It is now time to standardise! It has been taught to reproduce our output as closely as possible to the clean raw data. Following that is a feedforward deep neural network, followed by an output layer. The network parameters are changed to account for the difference between the input sample and the result. It is used to improve segmentation results using labelled image blocks. The first segmentation results come from putting each image block into a category based on where it is. Therefore, the portions may be labelled incorrectly. Non-cancer pixels are removed using threshold segmentation. As a result, this technique incorporates both open and closed morphological processing. Because of the distorted edges, there are fewer bulging borders and isolated zones. It will correct any picture flaws, such as concavities or holes, to improve the segmentation. It will use both morphological methodologies. The following is a summary of recent medical image segmentation research. A deep learning-based picture segmentation algorithm analysis is offered. Many authors investigated medical image segmentation issues. All the surveys mentioned above address deep neural networks. This study investigates these problems and the cutting-edge ways to solve them that have been proposed.

Deep learning is the foundational AI approach. Deep learning develops an artificial neural network by stacking data. The input, hidden, and output layers are being revealed [34]. These layers analyse signals while the hidden layers process them (shown in Figure 1).

**Figure 2:**
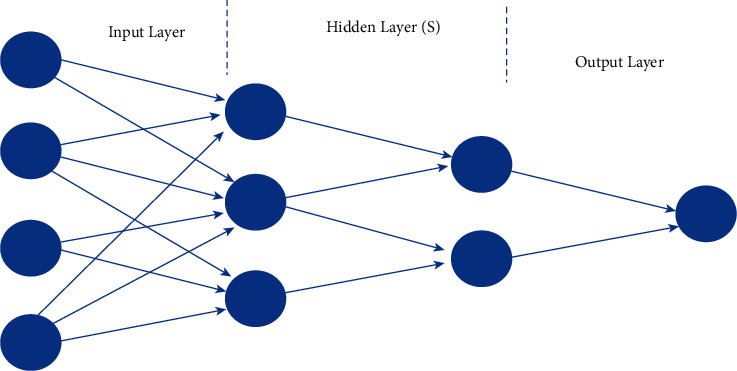
Model based on artificial neural networks (ANNs).

**Figure 3:**
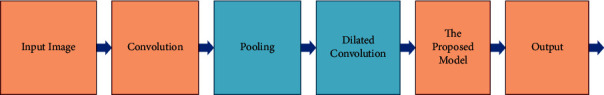
The design of a convolution neural network's architecture.

It describes the different deep learning neural networks that are used to segment the images.  Figure 2 shows that the following types of deep neural networks are often used to separate the parts of a picture. A CNN with three major neural layers is shown in Figure 3: convolutional, pooling, and fully linked [35]. A convolutional neural network is made up of three layers. The first layer of the algorithm is the convolutional layer. Layers provide a function. Edges and other visual components are recognised using convolution. Convolution is used to power several well-known image processing algorithms. These techniques can be divided into various subgroups. Convolution can be thought of as “multiplying” two arrays of numbers, each with a different width but the same dimensions. Because of this, a third integer array is made with the same size as the first two.

The convolution layer mathematically multiplies the near neighbours of a pixel. By convolutionally mapping the picture with its own kernels, CNN generates the feature maps. Therefore, the neural network's succeeding layers may process the input more effectively. This has no effect on the information. Subsampling is a technical term. With fewer layers, more layers may be computed. To generate high-level options, NN employs fully linked layers. Final conclusions are derived from the multiple replies in the input picture.

Important process blocks are interpolated between them. For each level of convolutions and pooling layers, the CNN models are often used in coding [36]. It asserts that the brain MRI images were classified using SqueezeNet and GoogleNet. Some of the drawbacks of CNN segmentation models are as follows: CNN's completely connected layers are incapable of handling high input amounts. Fully connected convolutional neural networks cannot segment objects because the number of relevant components fluctuates over time.

The structure of an FCN is made up of convolutional layers. The final, completely connected layer may be altered depending on the CNN system. With dense pixel-wise predictions, the [37] model can segment full-size images. It does this by upsampling and combining final layer feature maps with prior layer feature maps. A single model run yields complete segmentation. However, the standard FCN paradigm has flaws, including slowness and wasteful utilisation of global context information. Because FCN propagates across convolution and pooling layers, feature maps are downsampled. As a result, FCN predictions are low-resolution and object boundaries are blurry. Data points may be transported across domains using encoder-decoder models. This stage encodes the input *x* and predicts the output using the latent space representation as input. The following are encoder-decoder-based models for medical image segmentation:

The U-Net employs both downsampling and upsampling. Using three convolutions, the FCN downsampling architecture recovers features from downsampled input. Upsampling lowers the number of feature maps generated. When utilised as input for an upsample, they are maintained. Precision is possible with symmetric upsampling. Each pixel in the image is assigned its own segmentation map. U-Net modelling gives the following advantages: with just a few annotated training samples, the U-Net model can segment the images successfully. By downsampling and upsampling, the U-Net design expects an appropriate segmentation map. On the other hand, U-Net models are limited by: The image must be at least 572 pixels wide. Intermediate layers in deeper UNET models lose their learning rate, causing the network to ignore abstract information. Because of this, both the encoder and decoder networks build up feature mappings on the same scale.

**Figure 4:**
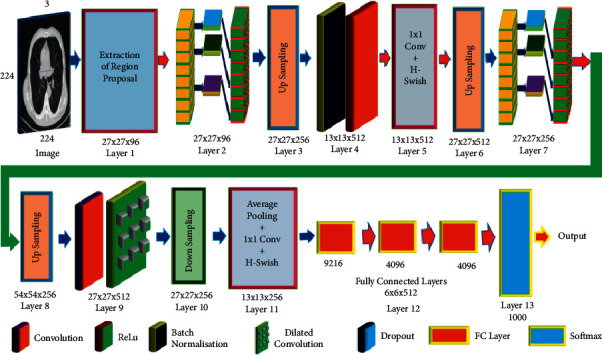
The proposed dilated ghost model.

It has also been used in the segmentation of medical pictures, the VNet has two components: compression and decompression. There are leftover convolution layers in the compression network. Convolution layers were created using volumetric kernels. Space represents assemblies and low-resolution feature maps. It is possible to achieve two-channel probabilistic segmentation in the front and back. RCNs are geographically connected neural networks. A convolutional network was used to do this; it uses a selective search R-CNN architecture to build bounding box region proposal networks. Using the region boundaries, CNN generates a feature vector map [38]. The visual properties required to categorise the objects in the area proposal network are included in the output dense layer. The system expects offset values to improve the accuracy of area or bounding box proposals.  Figure 4 depicts the proposed model. The model that has been suggested has 13 layers, and it extracts the finer features by making use of dilated convolution and max-pooling procedures. The ghost model eliminates the redundant characteristics, which simplifies the process and brings down the level of complexity. There are various faults with the RCN model: it cannot be used in real time since it must be trained on 2000 distinct domain ideas. It is only used once throughout the whole search. As a result, there is no learning, except negative candidate region recommendations.

**Figure 5:**
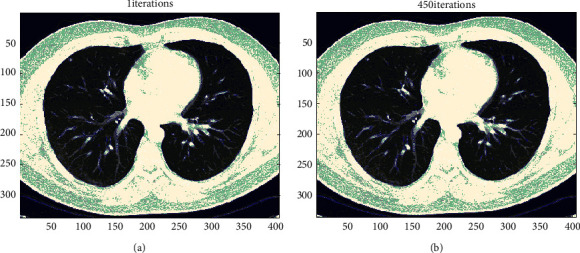
(a) A CT picture of the heart. (b) A CT scan of the heart.

**Figure 6:**
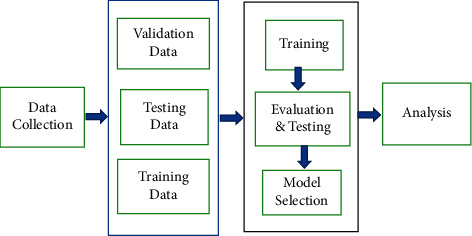
Training, testing, and validation process of the proposed model.

Figure 5 shows a picture of the heart and a segmented image. Deep neural networks are challenging to use for medical image segmentation [39, 40]. Aspects of dataset utilisation among the concerns with the dataset are annotated datasets of small size; they need a large amount of data to function properly. The training data has been well annotated. The dataset is used in many DL-based medicinal techniques. It is challenging to get large volumes of annotated medical images. Annotating fresh medical images takes both expertise and time. Many big databases are hosted on the Internet. The need for sophisticated datasets capable of training deep learning models and handling dense objects remains high. Because there are few synthetic 3D datasets, more complicated datasets are encouraged. Due to artifacts, intensity inhomogeneity, and other factors, automatically segmenting medical pictures is difficult.  Figure 6 depicts the fundamental framework of the model. When selecting a deep learning model, the body region to be segmented, imaging modalities, and disease kind must all be considered.

## 4. Results

The deep CNN used convolution, pooling, and normalisation to interpret the MR brain tissue images. Current medical image databases may be expanded by (a) conducting image enhancements such as rotating, flipping, cropping, and shearing, and (b) increasing the quantity of images. These improvements may boost the system performance. When data is unavailable, efficient model transfer learning may be applied. Finally, data from many sources is combined. Datasets that are unbalanced A class imbalance is seen in these datasets. For example, extremely variable data makes training DL models difficult, resulting in erroneous model outputs. This is because most people are healthy and reach their local minimum.

Recall, F1, accuracy, and precision are the metrics used to assess the machine learning performance. People who were classified as “positive” or “negative” (i.e., those who had heart disease or did not have heart disease) were correctly identified. Those who were mistakenly thought to be “negative” (having heart disease) or “positive” (not having heart disease) are shown in Table 1.

A class imbalanced dataset may be handled by (a) oversampling the data. Changing the performance assessment metric may aid in resolving the dataset imbalance problem. Existing data samples may be improved in certain circumstances. (d) Minority classes may be mixed together to correct for class imbalances in the dataset. Observations annotating 3D images takes time and is not always achievable. To label 3D data slices, partial labelling is employed. On 3D pictures with sparse annotation, a weighted loss function may be utilised to train a deep learning network. Because the weights of the unlabeled data are all zero, the model can only be trained with the tagged pixels.  Table 2 shows the *analysis of confusion measures for the purpose of applying a classifier: Predictive Negative (PN), Predictive Positive (PP), Actual Negative, and Actual Positive (AP)*.

Individual intensity variations of Photos with inhomogeneous colour and intensity are common. Gradient shading is caused via rasterization. Rendering is performed in rasterizing order based on the order of the layers in the Layers list. Rasterizing it may force it to relocate in order to match the layer order of the item receiving the shadow cast by the other object. To cast a shadow on a rasterized or flattened object, you may need to change the Layers list. It is capable of better segmenting the magnetic resonance images. TEM brightness variations are caused by inconsistent support films. As a result, segmentation takes a long time. Many nonparametric approaches are used to correct for intensity inhomogeneities. Prior to segmentation, prefiltering may reduce data inhomogeneities. Intensity inhomogeneities have been addressed through technological developments. Image texture that is Complex artefacts in medical photographs may result from image manipulation. Noise is produced by all of the sensors and electrical components used to gather images. It may have weak visual boundaries and grey levels that are too close together. Dermoscopic images may identify tissue overlap as well as small irregularities such as skin lines and hair. These limitations make specialising in medical imaging challenging. Images are cleaned up before segmentation to remove artefacts and noise. The image's noise is minimised while the image's limits and corners are preserved.

The following are significant challenges in training DNN for medical picture segmentation: not all models are created equal. Overfitting occurs when a model learns the subtleties and regularities of the training data better than the raw data. The model is trained with insufficient data [9]. Overfitting is reduced by increasing the dataset size and number of variables. Dropout techniques assist in minimising overfitting by rejecting part of the output of the network neurons each cycle. To avoid the model being very accurate, you may either halt the training process early, which is known as “early stopping,” or simplify the model by deleting less relevant inputs. These words allude to two distinct options: “early halting” and “early withdrawal.” If you stop the process too soon or skip too many steps, you may wind up with an underfitting model. To avoid underfitting, train your model for a long enough period of time or select input variables that are not critical enough to develop substantial linkages between the input and output variables. Both of these possibilities have the potential to produce incorrect results. Memory-saving algorithms in medicine need a great deal of memory. To be mobile phone compatible, the models' designs must be simplified. Simplicity and model compression may help to reduce DL model memory requirements. It is time to depart. Deep neural network training is time-consuming. Rapid deep neural network convergence is required for image segmentation.

Batch normalisation is one method of removing their values from the picture's average to discover pixels around zero. It is useful for quick information fusion. By adding pooling layers, it may also be possible to reduce the parameter dimension. Gradient fading intuitive deep neural networks have the problem of being unable to reverse the ultimate gradient loss through brain layers. In 3D models, the gradient problem is clear. This may be addressed in a variety of ways. (a) Deconvolution and SoftMax are used to upscale the gradient value of the intermediate hidden layer. (b) To increase the gradient value, auxiliary and buried layer losses are merged. To prevent vanishing gradients, appropriately initialise the network's weights. To be successful, these algorithms must be efficient. Extremely powerful CPUs and GPUs are needed. Some complex procedures may require the use of supercomputers to train the models. Due to these challenges, the researcher must use a limited set of criteria with low accuracy.

## 5. Conclusion

Deep convolutional neural networks are used in this study to categorise the items. Filter neurons that are driven by a target get feedback. The feedback mechanism of convolutional neural networks is known as feedforward composition. Researchers look at the feedback layer's effectiveness to improve the target neuron output. This technique maximises the input. It also discusses how to regain control after a loss. High-level semantics and visual space are combined in energy maps. It can recognise and segment photographs. This paper describes a method for segmenting medical images using feedback convolutional neural networks. The dependability and benefits of this technology are shown by comparing it to the best algorithms available through the CNN technique and the BRATS competition. To be effective, the suggested approach must be able to distinguish between picture components such as tumours and spines. We previously discussed the most extensively used deep learning-based medical image segmentation algorithms, as well as their advantages and disadvantages. There is also an explanation of the problems that come up when using deep networks to separate parts of medical images. Deep learning is gaining traction in picture segmentation. Deep neural network applications surpass classic image segmentation approaches. The findings of this research might aid in the development of neural network architectures for medical diagnosis. The researchers will also learn about current difficulties and solutions in medical image segmentation. This review study highlights significant literature and studies on the subject. Deep learning and machine learning have significantly improved image segmentation. They can segment many pictures. It aids with the identification of objects and illnesses in photographs. Future research might look at the processes given here and determine whether they work on various datasets. A future study could use publicly available datasets to compare the different deep learning algorithms that are already out there. Annotations on medical images, in particular, are the foundation of practically all deep learning systems. However, annotating medical data is not always achievable due to a number of factors, such as a lack of a skilled professional or an exceptionally rare condition. When there is a scarcity of large amounts of data, learning without supervision or with minimal monitoring may be advantageous. Consider how effective semi-supervised and unsupervised techniques will be in the medical business, as well as how to transition from supervised learning to transformational learning while retaining the same level of accuracy. Despite their greatest efforts, deep learning theories are still unable to supply all of the answers. Despite all of this effort, many questions remain unanswered. While this is true, we feel there is always a room for improvement.

## Figures and Tables

**Table 1:** Comparison of (I) accuracy, (II) precision, (III) recall, and (IV) F1-measure of various methods.

Methods	I	II	III	IV
Active appearance model	87.11	83.45	88.36	90.32
Support vector machine	96.23	89.78	96.67	94.71
RCNN	88.41	78.30	83.50	84.30
The proposed model	96.05	98.2	95.78	96.46

**Table 2:** Analysis of confusion measures for the purpose of applying a classifier predictive negative (PN), predictive positive (PP), actual negative, and actual positive (AP).

Method	Label	PN	PP
Active appearance model	AN	7425	7325
	AP	6187	6253

SVM	AN	6314	6014
	AP	5410	5142

RCNN	AN	5897	5001
	AP	4517	4221

The proposed model	AN	5794	5001
	AP	4876	4221

## Data Availability

A collection of images is taken for the research and it will be provided whenever required.
